# Multimodale Therapiekonzepte lokaler Verfahren mit der Immuntherapie: Rationale für kombinatorische Ansätze und mögliche klinische Therapiestrategien

**DOI:** 10.1007/s00120-022-01966-6

**Published:** 2022-11-22

**Authors:** Franziska Eckert

**Affiliations:** grid.22937.3d 0000 0000 9259 8492 AKH, CCC Wien, Klinik für Radioonkologie, Medizinische Universität Wien, Währinger Gürtel 18–20, 1090 Wien, Österreich

**Keywords:** Urogenitale Tumoren, Strahlentherapie, Oligometastasen, Immun-Checkpoint-Inhibitoren, „Stereotactic body radiotherapy“, Urogenital cancer, Radiotherapy, Oligometastases, Immune checkpoint inhibitors, Stereotactic body radiotherapy

## Abstract

Immuntherapien haben sich in der Behandlung onkologischer Erkrankungen i. Allg. sowie in der Uroonkologie etabliert. Die Rationale, diese Therapien mit Bestrahlung zu kombinieren, basieren auf den biologischen Effekten von Tumorbestrahlung, die weit über ein „physikalisches“ Abtöten von Tumorzellen hinausgeht. Abhängig von Dosis und Fraktionierung der Bestrahlung sowie dem verwendeten Tumormodell oder der Tumorentität können immunaktivierende und immunsupprimierende Effekte ausgelöst werden. Da eine Antitumorimmunantwort nicht lokal im Tumor sondern systemisch erfolgt, kann eine erfolgreich ausgelöste Antitumorimmunität in einer bestrahlten Metastase zu einem systemischen Ansprechen führen (abskopaler Effekt). In klinischen Studien werden Kombinationsschemata für lokal fortgeschrittene Tumorerkrankungen in der kurativen Situation zur Erhöhung der Heilungsraten sowie in der metastasierten Situation zur Verlängerung des Überlebens in der palliativen Situation eingesetzt. Ein weiteres Einsatzgebiet ist die lokale Behandlung von Oligometastasen oder in der Oligoprogression unter laufender Systemtherapie.

## Abstract (en)

Immunotherapy has been established in the treatment of cancer in general and specifically in uro-oncology. The rationale to combine these therapies with radiotherapy is based on the biological effects of irradiation, which exceed the “physical” properties of irradiation to kill tumor cells. Depending on radiation dose and fractionation as well as tumor model or tumor entity used, irradiation can lead to immune stimulation or immune inhibition. As immune response is not limited to the local tumor microenvironment but occurs on a systemic level, a successful anticancer immune response after irradiation of one metastasis can lead to systemic tumor control (abscopal effect). Clinical trials test combination regimens in locally advanced tumors in the curative setting to improve survival rates and in the metastatic setting to prolong survival in palliative treatment. In addition, radiotherapy is used for local treatment of oligometastases and oligoprogression under palliative systemic therapy.

Immuntherapeutische Ansätze gewinnen in der onkologischen Therapie mehr und mehr an Bedeutung. Nach den ersten Erfolgen in der Therapie metastasierter und lokal fortgeschrittener maligner Melanome ist die Immun-Checkpoint-Blockade in die Standardtherapie vieler Tumorentitäten integriert. Kombinatorische Ansätze mit lokalen Therapieverfahren, insbesondere Radiotherapie, erfordern ein Verständnis der zugrunde liegenden biologischen Mechanismen. Die synergistischen Effekte unterscheiden sich fundamental von Kombinationseffekten mit konventioneller Chemotherapie.

## Immuntherapie

### Entwicklung

Seit ca. 10 Jahren ergänzt die Immuntherapie, insbesondere die Immun-Checkpoint-Inhibition, die etablierten onkologischen Therapieverfahren (Operation, Strahlentherapie, Chemotherapie und zielgerichtete Therapie). Zu Beginn wurden Immun-Checkpoint-Inhibitoren (ICI, Antiköper gegen CTLA4 [„cytotoxic T‑lymphocyte-associated protein 4“] und PD1/PD-L1 [„programmed cell death protein 1/programmed cell death ligand 1“]) bei lokal fortgeschrittenen und metastasierten Melanomerkrankungen eingesetzt [1]. Die Patienten, die ein Ansprechen zeigten, erlangten oft über mehrere Jahre anhaltende Remissionen. Da konventionelle Chemotherapien bei metastasierten Melanomen nur sehr begrenzt Wirkung zeigten, waren diese Ergebnisse umso beachtlicher. Zwischenzeitlich hat sich die Immun-Checkpoint-Blockade in der palliativen sowie in der kurativen Therapie multipler Tumorerkrankungen etabliert [2].

Der biologische Hintergrund der Wirksamkeit ist, dass maligne Tumoren grundsätzlich ein immunsuppressives Tumormikromilieu bilden, wenn sie auf eine klinisch relevante Größe anwachsen. Einer der Mechanismen ist eine Überexpression von Immun-Checkpoints. Diese dämpfen bei Rezeptor-Liganden-Interaktion physiologischerweise das Immunsystem, um z. B. Autoimmunreaktionen zu verhindern. ICI sind blockierende Antikörper, die diese dämpfenden Signale unterdrücken und damit eine Aktivierung des Immunsystems bewirken. Dadurch wird das Immunsystem gegen den Tumor aktiviert. Dieser Mechanismus erklärt jedoch auch die möglichen Nebenwirkungen der Immun-Checkpoint-Blockade mit autoimmunähnlichen Phänomenen in nahezu allen Organsystemen. Neben der Immun-Checkpoint-Inhibition befinden sich weitere Immuntherapieklassen in der Entwicklung [3] und wurden teilweise für bestimmte Tumorentitäten bereits zugelassen (z. B. Anti-CD20-CAR-T-Zellen bei B‑Zell-Lymphomen und -Leukämien [4]).

### Uroonkologie

Immuntherapeutische Konzepte sind integraler Bestandteil der uroonkologischen Therapie geworden (zusammengefasst in [5]).

Die Immuntherapie ist in der Uroonkologie etabliert

Für metastasierende Urothelkarzinome wurden ICI für Platin-refraktäre Patienten und Patienten, die kein Platin erhalten können, in Studien geprüft und zeigten einen Überlebensvorteil gegenüber Taxan-haltigen Therapien. Nach Ansprechen auf eine Chemotherapie ergab sich ein signifikanter Vorteil für eine Immun-Checkpoint-Erhaltungstherapie. Im kurativen Setting zeigten immuntherapeutische Ansätze adjuvant nach neoadjuvanter Chemotherapie und Resektion einen Vorteil. Es wurden auch erste Studien zu ICI in der neoadjuvanten Situation durchgeführt. Zusätzlich werden ICI für nicht-invasive Blasenkarzinome eingesetzt, für die eine radikale Zystektomie nicht in Frage kommt.

Immuntherapien wurden für Nierenzellkarzinome schon sehr früh eingesetzt, da diese als sehr immunresponsive Tumoren gelten. Erste Studien wurden mit Interleukin 2 und Interferon durchgeführt. Die Therapie führte zu Remissionen, es traten jedoch hochgradige Nebenwirkungen auf. Studien in der metastasierten Situation erfolgten zumeist mit Kombinationen aus ICI und Tyrosinkinaseinhibitoren, dies wurde zur Standardtherapie. Auch in der adjuvanten Situation nach Nephrektomie bei Hochrisikopatienten führte Immun-Checkpoint-Inhibition zu einem signifikant verlängerten Überleben.

Die Datenlage für Prostatakarzinome und Peniskarzinome ist deutlich eingeschränkter. Für Prostatakarzinome ist die zelluläre Therapie mit Sipuleucel‑T zugelassen, wird jedoch nur begrenzt im klinischen Alltag eingesetzt. Der Einsatz von ICI wird v. a. für molekulare Subgruppen (z. B. Mismatch-repair-Defizienz) diskutiert. Für Peniskarzinome stehen aktuell nur Fallberichte zur Verfügung.

## Immuneffekte Bestrahlung

Bestrahlung ist keine rein „physikalische“ Behandlungsmodalität, die zu unkontrolliertem Zelltod führt, sondern hat multiple biologische Effekte intrazellulär in den Tumorzellen sowie im Tumormikromilieu. Immunologische Effekte einer lokalisierten Tumorbestrahlung beinhalten immunsuppressive sowie immunstimulatorische Mechanismen [6]. Diese Mechanismen sind auch abhängig von Bestrahlungsdosen und Fraktionierung. Immunstimulatorische Mechanismen beinhalten die Induktion von „immunogenem Zelltod“ (spezifische Arten von kontrolliertem Zelltod, der zu einer Aktivierung des angeborenen Immunsystems führt), gesteigerte T‑Zell-Infiltration in den Tumor und die Aktivierung des Immunsystems durch intrazellulär-zytoplasmatische Freisetzung doppelsträngiger DNA. Neben diesen „positiven“ Effekten, die in den letzten Jahren zur Entwicklung multimodaler Therapiekonzepte führten, hat die Bestrahlung von Tumoren jedoch auch immunsuppressive Effekte im Tumormikromilieu. Neben einer Anreicherung immunsuppressiver regulatorischer T‑Zellen (Treg-Zellen) wird auch eine immunsuppressive Population von Makrophagen (M2-Polarisierung) vermehrt in bestrahlten Tumoren gefunden. Zusätzlich werden Immun-Checkpoints wie PD-L1 vermehrt in bestrahlten Tumoren exprimiert.

Eine Bestrahlung kann Antitumorimmuneffekte verstärken oder auch abschwächen

Wird durch eine lokale Strahlentherapie eine effiziente Immunantwort ausgelöst, kann dies auch ohne systemische Therapie zu einem systemischen Tumoransprechen führen. Dies ist zu erklären, da eine adaptive Immunantwort nicht begrenzt auf das lokale Tumormikromilieu stattfindet. Im Tumor werden Antigene präsentiert, bei gleichzeitiger Aktivierung antigenpräsentierender Zellen (z. B. dendritischer Zellen) migrieren diese in die drainierenden Lymphknoten. Dort werden über CD4^+^-Helferzellen zytotoxische CD8^+^-T-Zellen, die die präsentierten Antigene spezifisch erkennen, zu klonaler Expansion aktiviert. Diese Zellen gelangen in den Blutkreislauf und somit systemisch in alle Organsysteme. Um in den Tumor zu gelangen, verlassen diese Zellen die Blutbahn, im Tumormikromilieu können zytotoxische T‑Zellen Tumorzellen direkt lysieren [7]. Da Immunzellen systemisch in den Blutkreislauf gelangen, können auch nicht bestrahlte Metastasen durch aktivierte zytotoxische T‑Zellen zur Regression gebracht werden.

## Abhängigkeit von Bestrahlungsmodalitäten

Diese Mechanismen sind abhängig von den untersuchten Modellen/Tumortypen, dem bestrahlten Volumen sowie den Bestrahlungsdosiskonzepten. Für verschiedene Schritte der Antitumorimmunantwort werden verschiedene optimale Dosiskonzepte beschrieben. Für die Induktion von immunogenem Zelltod werden hohe Einzeldosen als optimal beschrieben, da hierdurch vorrangig nekrotische und nekroptotische Formen des Zelltods ausgelöst werden, die als immunogener eingestuft werden als apoptische Zelltodformen. Für moderat hypofraktionierte Konzepte mit höheren Einzeldosen wurden in einem Mausmodell eines syngenen Mammakarzinoms mit Bestrahlung in Kombination mit CTLA-4-Antikörpern abskopale Effekte beschrieben [8]. Andererseits wurde für diese Dosisstufen auch eine Anreicherung immunsuppressiv wirkender M2-polarisierter Makrophagen erkannt. Für eine Bestrahlungsdosis von 2 Gy, wie sie für normofraktionierte Konzepte in der Klinik eingesetzt wird, wurde ein positiver Einfluss auf die T‑Zell-Infiltration ins Tumorgewebe beschrieben.

Zusammenfassend ist kein „optimales“ Bestrahlungsregime auszumachen, das alle immunonkologischen Effekte gleichermaßen positiv beeinflusst. Je nach dominantem immunonkologischem Mechanismus in einem speziellen Tumortyp und selbst patientenindividuell müssten die Bestrahlungskonzepte idealerweise abgestimmt werden. Es werden auch Überlegungen angestellt, verschiedene Bestrahlungsdosen zu kombinieren, z. B. eine hohe Einzeldosis auf den Tumor/einen Tumorteil zu Beginn um immunogenen Zelltod auszulösen mit hypofraktionierten Schemata zur Induktion eines abskopalen Effekts.

## Abskopaler Effekt

Der abskopale Effekt wird mit Strahlentherapie alleine in der Klinik nur sehr selten beobachtet. Mit der Einführung der Immuntherapie in die klinische Routine nahmen diese Fallberichte zu, als zugrunde liegende Mechanismen konnten Antitumorimmunreaktionen identifiziert werden [9]. Dies führte zur Planung multipler Studien, die in der metastasierten Tumorsituation die Kombination von ICI mit lokaler Bestrahlung einzelner Metastasen im Hinblick auf eine systemische Tumorkontrolle evaluieren. Hierbei ist entscheidend, abskopale Effekte richtig zu definieren.

Abskopaler Effekt ist ein immunvermittelter, systemischer Antitumoreffekt einer lokalen Strahlentherapie

Es handelt sich um Effekte außerhalb des bestrahlten Volumens, die über den alleinigen systemischen Effekt der Immuntherapie hinausgehen [10]. Bei gleichzeitigem Start von Bestrahlung und Immuntherapie kann dies nur im randomisierten Setting belegt werden, wenn im Kombinationsarm mehr systemische Tumorregressionen auftreten.

## Kombinationstherapie in der Klinik

Die ersten klinischen Studien zu Kombinationstherapien schlossen metastasierte Patienten ein, bei denen zusätzlich zur Immuntherapie eine oder mehrere Metastasen bestrahlt wurden. Oft wurden stereotaktische, hypofraktionierte Bestrahlungskonzepte verwendet, die lediglich makroskopischen Tumor im Hochdosisbereich eingeschlossen hatten. Zunehmend wurden ICI in kurative radioonkologische Konzepte integriert. In der PACIFIC-Studie wurde anschließend an eine kurative Radiochemotherapie bei Patienten mit lokal fortgeschrittenen Lungenkarzinomen eine Erhaltungstherapie mit Durvalumab durchgeführt. Verglichen mit dem Kontrollarm zeigte sich ein signifikant verlängertes krankheitsfreies Überleben sowie Gesamtüberleben [11]. Dieser Erfolg zeigte sich jedoch nicht in allen Tumorentitäten. Für Plattenepithelkarzinome im Kopf-Hals-Bereich zeigte sich in randomisierten Studien kein Vorteil für die Hinzunahme von ICI zur Standardradiochemotherapie. Auch für Glioblastome waren die randomisierten Studien negativ. Dementsprechend müssen in weiteren translationalen Projekten die besten Modalitäten für Kombinationstherapien sowie mögliche prädiktive Biomarker weiter entwickelt werden.

**Figure 1:**
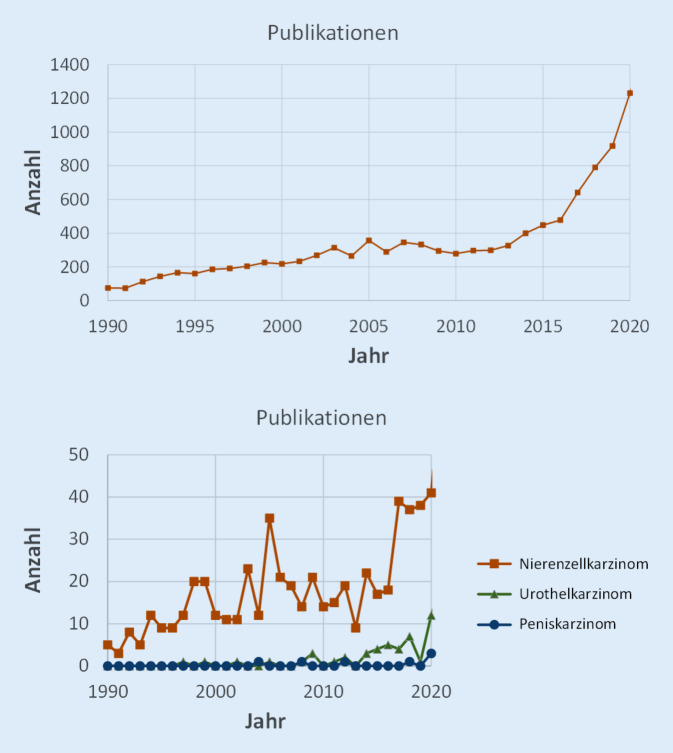

Die zunehmende Bedeutung der Kombination von Immun- und Strahlentherapie ist an den steigenden Zahlen veröffentlichter Artikel in der Onkologie allgemein sowie auch für uroonkologische Tumorentitäten zu sehen (Abb. 1). Laufende Studien im uroonkologischen Bereich sind in Tab. 1 zusammengefasst.

**Table 1:** 

	Titel der Studie	Stadium	Strahlentherapie	Immuntherapie	Phase	Studien-Nummer
Nierenzellkarzinom	Testing the addition of stereotactic radiation therapy with immune therapy for the treatment of patients with unresectable or metastatic renal cell cancer	Irresektabel oder metastasiert	SBRT	Avelumab, Ipilimumab, Nivolumab, Pembrolizumab	2	NCT05327686
	Study assessing stereotactic radiotherapy in therapeutic strategy of oligoprogressive renal cell carcinoma metastases	Metastasiert, oligoprogressiv	SBRT	Nicht spezifiziert	2	NCT04299646
	SBRT with combination ipilimumab/nivolumab for metastatic kidney cancer	Metastasiert	SBRT	Ipilimumab/Nivolumab	2	NCT04090710
Urothelkarzinom	Radio-immunotherapy before cystectomy in locally advanced urothelial carcinoma of the bladder	Lokal fortgeschritten	Normofraktionierte Strahlentherapie der Blase und des Beckens	Nivolumab	2	NCT03529890
	Neutron radiation therapy and pembrolizumab in treating participants with advanced urothelial carcinoma	Fortgeschritten	Neutronen Bestrahlung	Pembrolizumab	2	NCT03486197
	Immunotherapy with or without radiation therapy for metastatic urothelial cancer	Metastasiert	SBRT	Atezolizumab	2	NCT04936230
	A study of chemotherapy and radiation therapy compared to chemotherapy and radiation therapy plus medi4736 (durvalumab) immunotherapy for bladder cancer which has spread to the lymph nodes (the inspire study)	Metastasiert	SBRT	Atezolizumab	2	NCT04936230
	Avelumab and radiation in muscle-invasive bladder cancer	Lokal fortgeschritten (Platin-unfähig)	Standardstrahlentherapie Blase/Becken	Avelumab	2	NCT03747419
	Chemoradiotherapy with or without atezolizumab in treating patients with localized muscle invasive bladder cancer	Lokal fortgeschritten	Normofraktionierte Radiochemotherapie (Gemcitabin oder Cisplatin oder 5‑FU und MMC)	Atezolizumab	3	NCT03775265
	Neoadjuvant sasanlimab with radiation as an in situ vaccine for cisplatin-ineligible muscle invasive bladder cancer	Lokal fortgeschritten	SBRT neoadjuvant vor Zystektomie	Sasanlimab	2	NCT05241340
	Radiation and durvalumab immunotherapy as neoadjuvant treatment for MIBC	Lokal fortgeschritten	Neoadjuvant 1 × 8 Gy	Durvalumab	2	NCT04543110
Prostatakarzinom	Phase 3 study of ProstAtak® immunotherapy with standard radiation therapy for localized prostate cancer	Lokalisiert	Kurative Standardstrahlentherapie	Aglatimagene besadenovec + Valaciclovir	3	NCT01436968
	Combination of nivolumab immunotherapy with radiation therapy and androgen deprivation therapy	Lokalisiert	Kurative Strahlentherapie + HDR-Brachytherapie + ADT	Nivolumab	1/2	NCT03543189
	T‑cell clonality after stereotactic body radiation therapy alone and in combination with the immunocytokine M9241 in localized high- and intermediate-risk prostate cancer treated with androgen deprivation therapy	Lokalisiert	Strahlentherapie + ADT	M9241	2	NCT05361798
	Prostate Cancer with oligometastatic relapse: combining stereotactic ablative radiotherapy and durvalumab (MEDI4736)	Oligometastatisch	SBRT	Durvalumab	2	NCT03795207
Peniskarzinom	Penile cancer radio- and immunotherapy clinical exploration study	Lokoregional/metastasiert (stratifiziert)	Strahlentherapie des Beckens	Atezolizumab	2	NCT03686332

*SBRT* „stereotactic body radiotherapy“, *MMC* Mitomycin C, *ADT* Androgendeprivationstherapie, *HDR* „high dose rate“, *5‑FU* 5‑Fluorouracil

### SBRT („stereotactic body radiotherapy“)

**Figure 2:**
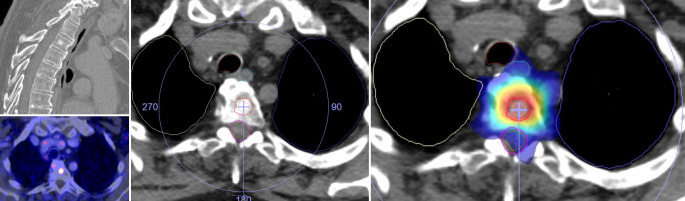

Eine weitere klinische Situation, in der eine Immuntherapie (oder auch zielgerichtete Therapien) mit einer Strahlentherapie kombiniert wird, ist die oligometastasierte Situation oder eine Oligoprogression unter Systemtherapie. In diesen Situationen werden zunehmend lokaltherapeutische Maßnahmen für die wenigen vorhandenen oder unter Systemtherapie progredienten Metastasen eingesetzt. Neben chirurgischen Methoden kommen stereotaktische ablative Strahlentherapieverfahren zur Anwendung. Hierbei werden kleine Volumina, die in der Regel nur die makroskopisch sichtbaren Metastasen umfassen, konformal behandelt. Dadurch ist es möglich, die Strahlentherapie in wenigen Fraktionen mit hohen Einzeldosen durchzuführen. Die lokalen Kontrollraten entsprechen chirurgischen Metastasenresektionen. Ein Beispiel einer stereotaktischen Bestrahlung ist in Abb. 2 gezeigt. Für Patienten mit metastasierten Lungenkarzinomen konnte durch die Hinzunahme stereotaktischer Bestrahlung zur Systemtherapie ein signifikant verlängertes Gesamtüberleben erreicht werden [12].

Durch die Lokaltherapie der progredienten Herde werden Klone an Tumorzellen entfernt

Durch die Lokaltherapie einzelner oder weniger progredienter Metastasen unter Systemtherapie kann die an den anderen Metastasen wirksame Systemtherapie fortgeführt werden. Die biologische Rationale für die Lokaltherapie der progredienten Herde ist, dass dadurch Klone an Tumorzellen entfernt werden, die z. B. durch Mutationen Resistenzen gegen die laufende Systemtherapie entwickelt haben. Für den Einsatz einer ablativen Strahlentherapie ergibt sich die zusätzliche Rationale eines möglichen abskopalen Effekts.

**Figure 3:**
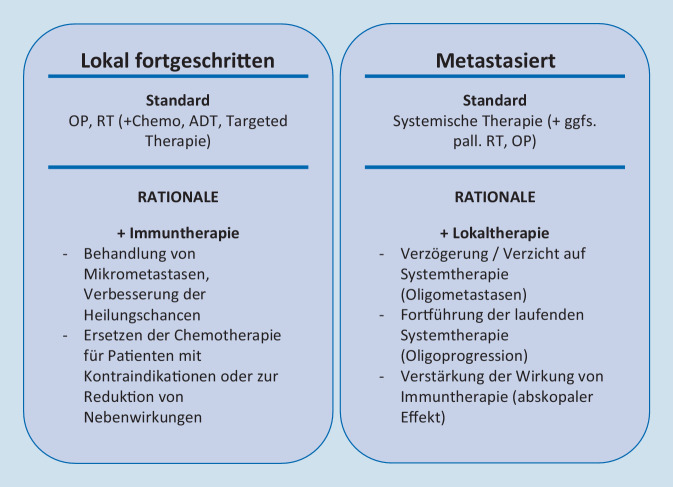

Zusammengefasst ergibt sich eine Rationale für die Kombination von Strahlentherapie mit Immuntherapien für lokal fortgeschrittene Tumoren in kurativer Therapieintention sowie in der metastasierten Situation (Abb. 3).

## Fazit für die Praxis

- Immuntherapien haben einen festen Platz in der Behandlung uroonkologischer Erkrankungen.
- Strahlentherapie löst vielfältige Immuneffekte im Tumor und systemisch aus.
- Die Kombination aus Strahlentherapie und Immuntherapie kann in der lokal fortgeschrittenen und der metastasierten Situation sinnvoll sein und wird in klinischen Studien geprüft.
- Die Hinzunahme von lokaler Bestrahlung zur Immuntherapie kann eine systemische Tumorkontrolle auslösen (abskopaler Effekt).
- Hochdosierte stereotaktische Bestrahlung wird bei Oligometastasierung und Oligoprogression eingesetzt.
